# The Functional Study of the N-Terminal Region of Influenza B Virus Nucleoprotein

**DOI:** 10.1371/journal.pone.0137802

**Published:** 2015-09-14

**Authors:** Ming Liu, Mandy Ka-Han Lam, Qinfen Zhang, Ruth Elderfield, Wendy S. Barclay, Pang-Chui Shaw

**Affiliations:** 1 Centre for Protein Science and Crystallography, School of Life Sciences, The Chinese University of Hong Kong, Shatin, N.T., Hong Kong, China; 2 State Key Laboratory of Biocontrol, School of Life Sciences, Sun Yat-sen University, Guangzhou, China; 3 Section of Virology, Faculty of Medicine, Imperial College London, London, United Kingdom; US Food and Drug Administration, UNITED STATES

## Abstract

Influenza nucleoprotein (NP) is a major component of the ribonucleoprotein (vRNP) in influenza virus, which functions for the transcription and replication of viral genome. Compared to the nucleoprotein of influenza A (ANP), the N-terminal region of influenza B nucleoprotein (BNP) is much extended. By virus reconstitution, we found that the first 38 residues are essential for viral growth. We further illustrated the function of BNP by mini-genome reconstitution, fluorescence microscopy, electron microscopy, light scattering and gel shift. Results show that the N terminus is involved in the formation of both higher homo-oligomers of BNP and BNP-RNA complex.

## Introduction

The annual outbreaks of influenza have posed heavy burdens to global public health. While influenza A has drawn much of the attention, the threats from influenza B cannot be underestimated. Influenza B viruses has been involved in 16 epidemics in the past 70 years with high morbidity and mortality. It has taken a considerable proportion in the flu season especially in children [1,2] and it also has a lower sensitivity to neuraminidase inhibitors [3,4,5]. Therefore it is important to illustrate details of influenza B virus functions.

The genome of influenza B virus comprises eight negative-sense RNA segments encoding 11 polypeptides, including RNA polymerase components PA, PB1 and PB2, haemagglutinin (HA), nucleoprotein (NP), neuraminidase (NA), membrane protein NB, matrix protein M1, ion channel BM2, non-structural proteins NS1 and NS2[6]. Among these proteins, NP is the major component of the ribonucleoprotein (RNP), which is a complex consisting of RNA, NP and RNA polymerase and has the role in transcription and replication of the viral genome [7,8,9].

The crystal structure of BNP displays a crescent shape that contains a head domain and a body domain. In between the two domains is a deep groove enriched for basic amino acid residues that facilitates RNA binding [10]. BNP forms an oligomer by inserting the tail-loop into the tail-loop binding groove of the neighboring molecule. The structures of ANP and BNP are grossly similar. The root-mean-square deviation of backbone atoms in secondary structure regions is 1.457 Å between FluA H1N1 NP and FluB NP. As a result, the two proteins have comparable secondary structure elements and similar α-helices and β-strands [10,11,12]. However, the two proteins bear a significant variation at the N-terminal region, with BNP significantly longer (71 aa) than that of ANP (21 aa) and there is little homology between the two. The structures of this region in both ANP and BNP are disordered [10,11,12].

It is known that the N-terminal of BNP plays a role in the transcription and replication of viral RNP, possibly by nuclear targeting (aa 44–47) and proteomic protection (aa 1–15)[13,14]. However, whether it is involved in other major functions of BNP, such as oligomerization or RNA binding, have not been explored. To obtain a more complete understanding on the role of this region in BNP, we set forth to analyze the function, with special attention on its role in oligomerization, nuclear import, RNA binding and viral growth.

## Materials and Methods

### Biological materials

**Fig 1:**
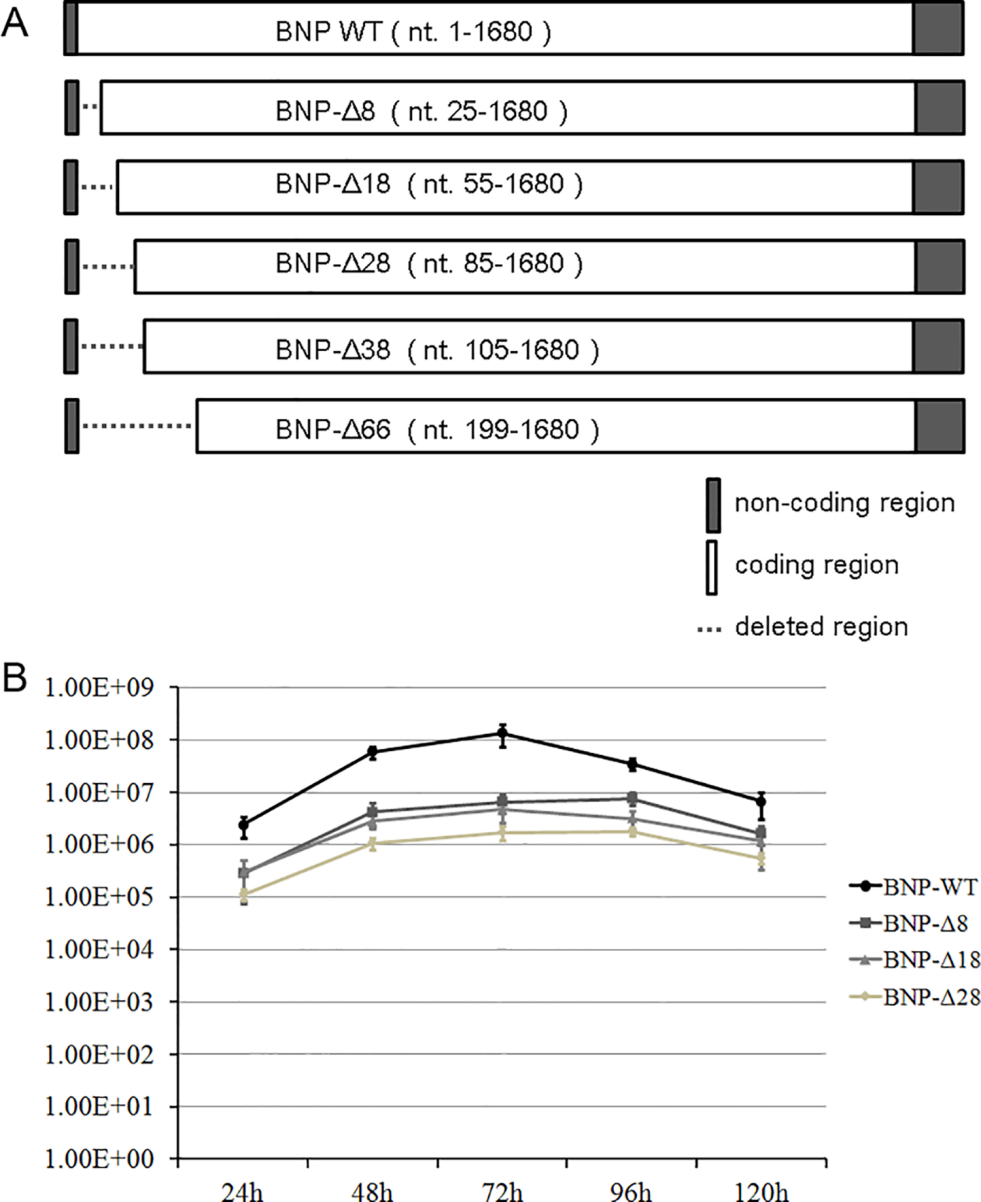
The construction and growth curves of influenza B virus with BNP variants. (A) Construction of influenza B NP mutants by systematically deleting the N-terminal coding region of NP. (B) Growth curves of influenza B wild-type and NP N-terminal deletion mutants after virus rescue. The growth curves are averages from three independent replicates.

Hela cells(ATCC CCL2)[15], Madin-Darby canine kidney (MDCK) cells (ATCC CCL-34) [14,16,17] and HEK 293T/17 cells (ATCC CRL-11268)[10,11] were maintained in Dulbecco’s modified Eagle’s medium supplemented with 10% fetal calf serum. All cells were incubated at 37°C in 5% CO2. Plasmids pCIPA, pCIPB1 and pCIPB2 expressing RNA polymerase subunits of influenza B/Panama/45/90 virus were described previously [10,11]. Plasmid pPol-Luci-NA-RT was constructed with the non-coding sequence of NA segment flanking the firefly luciferase coding sequence. The nucleoprotein (B/panama/45/1990) was used as the template for BNP cloning. Wild-type sequence and mutants were cloned into pcDNA3 (Life technologies), pEGFP-N1 (Clontech) and pRHisMBP (from K. B. Wong, the Chinese University of Hong Kong). Wild-type and mutant viruses were generated using a reverse genetic system as previously described [16]. Plasmids for rescue of influenza B virus (pCIPA, pCIPB1, pCIPB2, PCINP, PB1, PB2, PA, M, NS, HA, NA and NP) were transfected to 293T cells for virus constitution as previously described [16,17]. Deletion of the first 8, 18, 28, 38 and 66 aa of the NP gene was achieved by removing the corresponding nucleotide sequences and inserting ATG at the start of coding regions, as shown in Fig 1A. The transfected 293T cells were incubated with MDCK cells for 3–7 days at 24 hours post-transfection. Viral RNA was extracted using the QIAamp viral RNA kit (QIAGEN), reverse-transcribed and sequenced to confirm if the virus carried the mutations.

### Virus replication kinetics

MDCK cells were infected with either wild-type or mutated viruses at an MOI of 0.001. The cell supernatant was collected at 24 h, 48 h, and 72 h post-infection. The viral concentration was determined by plaque assay on MDCK cells.

### Plaque assays

MDCK cells were infected with 10-fold dilutions of each virus for 1 h at 37°C. After that the cells were overlaid with 0.5% agar in DMEM with 1 μg/ml TPCK trypsin and incubated at 37°C for 72 h. Cells were stained with coomassie blue. Viral plaques were counted to determine viral concentration.

### Mini-genome reconstitution assay

Plasmid pCIPA, pCIPB1, pCIPB2, pcDNA-BNP (wild type or NP mutants), and pPol-Luci-BNA-RT (0.125 μg each, except the amount of pcDNA-BNP mutants was individually adjusted to have a similar expression level to the wild-type) were co-transfected to 1x10^5^ 293T cells for RNP complex reconstitution. Plasmid for enhanced green fluorescent protein (EGFP) expression (0.06 μg) was also co-transfected for normalization. Plasmid pCIPA, pCIPB1, and pCIPB2 expressing RNA polymerase subunits of influenza B/Panama/45/90 virus were described previously. Plasmid pPolI-Luc-RT (for the generation of pPol-Luci-BNA-RT) and pEGFP were provided by Dr. L. L. M. Poon of the University of Hong Kong. For the negative control, empty pcDNA plasmid instead of pcDNA-BNP was transfected to cells. At 48 h post-transfection, 293T cells were lysed by Steady-Glo assay reagent (Promega) for 5 min followed by luminescence measurement on a luminometer (Victor2 1420 multilabel counter; Wallac) according to the manufacturer’s instructions. The statistic parameters were calculated by GraphPad Prism using T-test method.

### Expression and purification of BNP

MBP-tagged BNP variants were expressed in *Escherichia coli* C41 (DE3). The cells were lysed in 20 mM sodium phosphate, 150mM NaCl, pH 6.5. The lysate was passed through an amylose column (New England BioLabs, Ipswich, MA). The bound protein was eluted in buffer containing 20 mM maltose. The eluate was incubated with thrombin (100 U) (Sigma-Aldrich) and RNase A (300 U) (Sigma-Aldrich) at 4°C overnight to remove the MBP tag and RNA from BNP. It was then passed through a heparin high-performance (HP) column (GE Healthcare, Waukesha, WI). NP was eluted with a 0 to 1.5 M NaCl gradient in the same buffer.

### Static light scattering

Wild-type or variant BNP proteins were subjected to static light scattering using a miniDAWN triangle (45°, 90°, and 135°) light scattering detector (Wyatt Technology, Santa Barbara, CA) connected to an Optilab DSP interferometric refractometer (Wyatt Technology Corporation, Santa Barbara, CA). This system was connected to a Superdex 200 column (GE Healthcare) controlled by a BioLogic DuoFlow chromatography systems. Before sample injection, the miniDAWN detector system was equilibrated with 100 mM sodium phosphate (pH 6.0) and 100 mM NaCl for at least 2 h to ensure a stable baseline signal. The flow rate was set to 0.5 ml/min, and the sample volume was 100 μl. The laser scattering (687 nm) and the refractive index (690 nm) of the respective protein solution were recorded. Wyatt ASTRA software was used to evaluate the obtained data.

### Electron Microscopy (EM)

EM samples of NP with or without RNA (RNA sequence: 5’-GGG AGA UUU UUU UUU UUU UUU UUU UUU UUU UUU UUU UUU UUU UUU UUU UUU-3’ (RiboBioscience, Guangzhou, China)) were prepared by conventional negative staining. 5 μl sample was applied to a glow-discharged carbon-coated EM grid. After 1 min incubation, the grid was washed with two drops of water and stained with two drops of 0.75% (w/v) uranyl formate. Grids were examined with a JEOL JEM-100CX II electron microscope equipped with a tungsten filament and operated at an acceleration voltage of 100 kV. Images were recorded with a CCD camera.

### Gel shift assay

A 24-nucleotide (nt) 2-O-methylated RNA oligonucleotide with the sequence 5’-UUU GUU ACA CAC ACA CAC GCU GUG-3’ (RiboBioscience, Guangzhou, China) (10 μM) was incubated with an increasing amount of purified wild-type NP or protein variants (0, 5, 10, and 20 μM) for 30 min at room temperature. The mixture was resolved by agarose gel electrophoresis and visualized by ethidium bromide staining.

### Detection of GFP-fusion protein by direct fluorescence

Hela cells were seeded on a coverslip at a density of 6x10^4^ cells per ml. Cells were transfected individually with plasmid DNA encoding GFP-fusion protein after 18–24 h. At 24 h post-transfection, the cells were washed by PBS for three times and fixed with 4% paraformaldehyde in PBS for 10 min at room temperature. The fixed cells were washed by PBS for three times and stained by DAPI for 2 min at room temperature. The coverslip was mounted and visualized with a fluorescence microscope.

## Results

### The N-terminal region of BNP is essential for the growth, replication and transcription of influenza B virus

To evaluate the importance of the N-terminal of NP in virus growth, we attempted to rescue recombinant influenza B viruses based on influenza B/Florida/04/2006 by reverse genetics. These mutants carried deletion for residues at the N terminal of NP (Flu B-BNP-Δ8, Flu B-BNP-Δ18, Flu B-BNP-Δ28, Flu B-BNP-Δ38 and Flu B-BNP-Δ66). For the last two mutants bearing deletions of 38 or 66 residues, no virus was reconstituted. This suggested that lengthy deletion had an adverse effect on the survival of the virus. It is known that 60 nucleotides at the 3’ end and 120 nucleotides at the 5’ end in the coding region are sufficient for the packaging of influenza A NP genome [18]. Whether the truncated nucleotide sequence contained any cis-packaging signal cannot be concluded because of the poor sequence homology between influenza A and influenza B NP at the 3’ end. Recombinant viruses were rescued with deletions of up to 28aa, and replication was compared under multicycle conditions in MDCK cells infected at an MOI of 0.001. The virus harvested at every 24 h till 120h post infection was titrated by plaque assay. Deletion of even 8aa from the BNP N terminus resulted in retardation of viral growth and decrease in titer (Fig 1B). Further truncations of 18 or 28 aa also resulted in attenuated viruses but with little further decrease in titre over the 8aa truncation. We notice Sherry and collaborators also systematically truncated the N-terminal of NP in the B/Yamanashi/98 system [14]. They retained the putative packaging signals by inserting novel ATG initiation codons within the coding region of NP. However they were only able to reconstitute viral variants with NP truncated up nine amino acids. This difference in the length of flu B NP that is required for virus reconstitution may be due to the difference of the rescuing system and plasmid construction. Nevertheless, the work of Sherry and collaborators also implicates the N-terminal region of NP in the transcription and replication of the virus.

**Fig 2:**
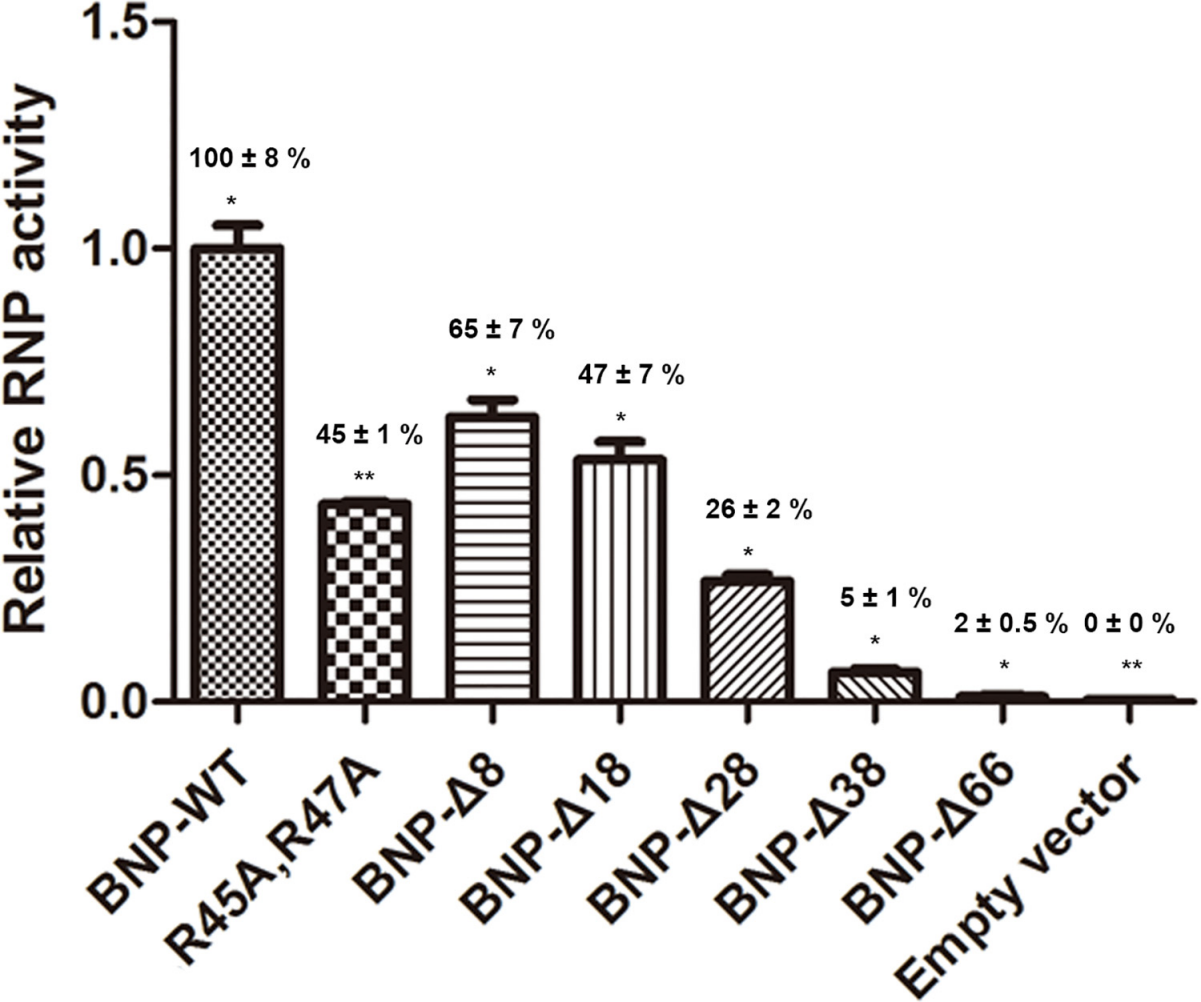
The viral RNP activity of wild-type BNP and variants. Viral RNP activity of wild-type and BNP N-terminal deletion variants. For each mutants triplicate wells were set and recorded. Experiments are repeated twice. P values are calculated by T-test algorithm. *, *P* < 0.01; **, *P* < 0.001.

We next used mini-genome reconstitution assay to verify whether truncation of the N terminus affects the functions of vRNP. As shown in Fig 2, deleting first 8, 18, 28 resulted in a serial reduction in RNP activities, while truncation of 38 or 66 aa substantially reduced the RNP signal to only 5% or 2% of the activity obtained with full length NP. This implied a functional role of aa 1–66 for the transcription and replication of vRNP. The deleterious effect on RNP activities may explain the growth retardation of failure to reconstitute the recombinant virus mutants.

### The first 38 residues carry functions other than NLS

**Fig 3:**
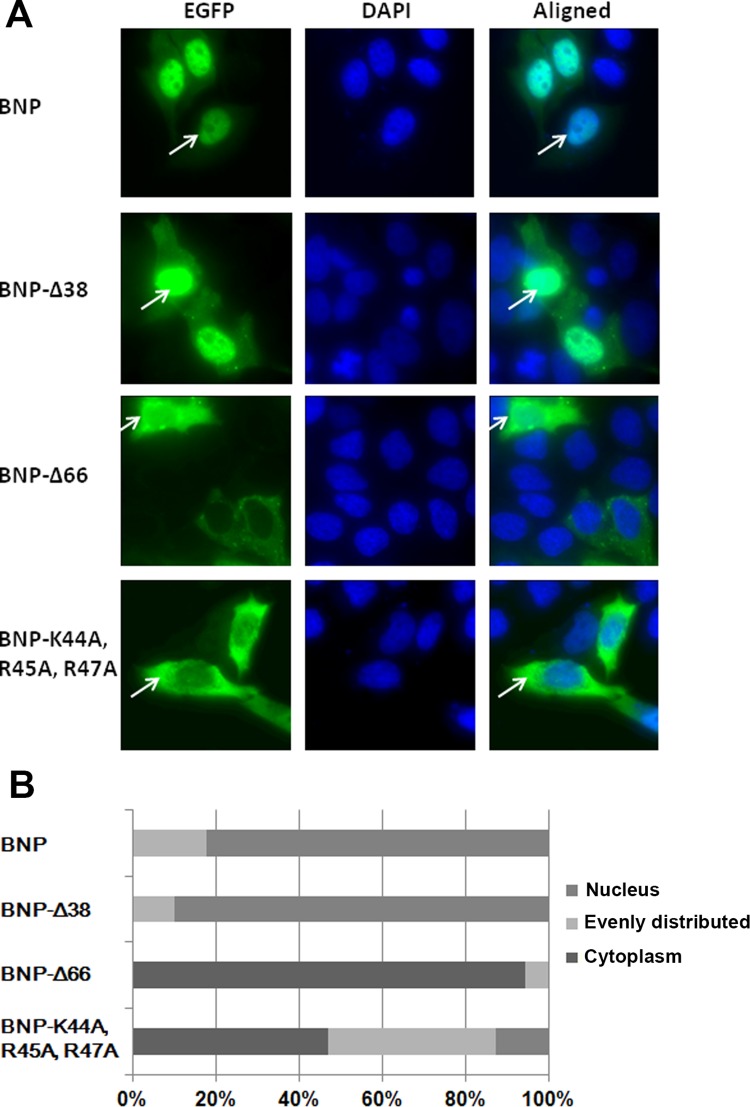
The nuclear localization of wild-type BNP and variants. **(A)** Inmmuno-fluorescence images of BNP-GFP chimeric constructs. The subcellular location was indicated by the EGFP signal (green). The nucleus was stained by DAPI (blue). The locations of BNP were shown by white arrows. BNP and BNP-Δ38 mainly located in the nucleus, while BNP-K44A, R45A, R47A and BNP-Δ66 stayed predominantly in the cytoplasm. (B) For each construct apppoximately 50 cells were counted to generate the subcellular distributions. GFP signals overlapped with DAPI staining was characterized as nuclear localized, otherwise it was characterized as cytoplasmic localized. Subcellular localization of GFP was sorted as predominantly cytoplasmic, evenly distributed and predominantly nuclear.

Previous studies have found that a non-conventional nuclear localization signal (NLS) K_44_RTR_47_ was located at the N-terminal of BNP [13,14]. To further describe the role of this NLS, BNP constructs, including Wild-type BNP, BNP with deletion of aa 1–38 (BNP-Δ38), aa 1–66 (BNP-Δ66), and BNP NLS mutant (BNP-K44A, R45A, R47A) were fused with Green fluorescent protein (GFP) and their subcellular localization visualized by fluorescent microscopy. As shown in Fig 3A and 3B, wild-type NP and the 1–38 deletion variant (BNP-Δ38) were mainly retained in the nucleus in a comparable proportion, which indicates that the removal of aa 1–38 did not hinder the nuclear entry of NP. In contrast, the GFP-BNP-K44A, R45A, R47A and 1–66 deletion variant (BNP-Δ66) stayed predominantly in the cytoplasm. The vRNP activity with the BNP-K44A, R45A, R47A mutant was only 45% of the wild-type (Fig 2). This indicated the NLS in N-terminal has a considerable effect on the RNP function.

### The N-terminal region of BNP helps to retain the oligomeric structure

**Fig 4:**
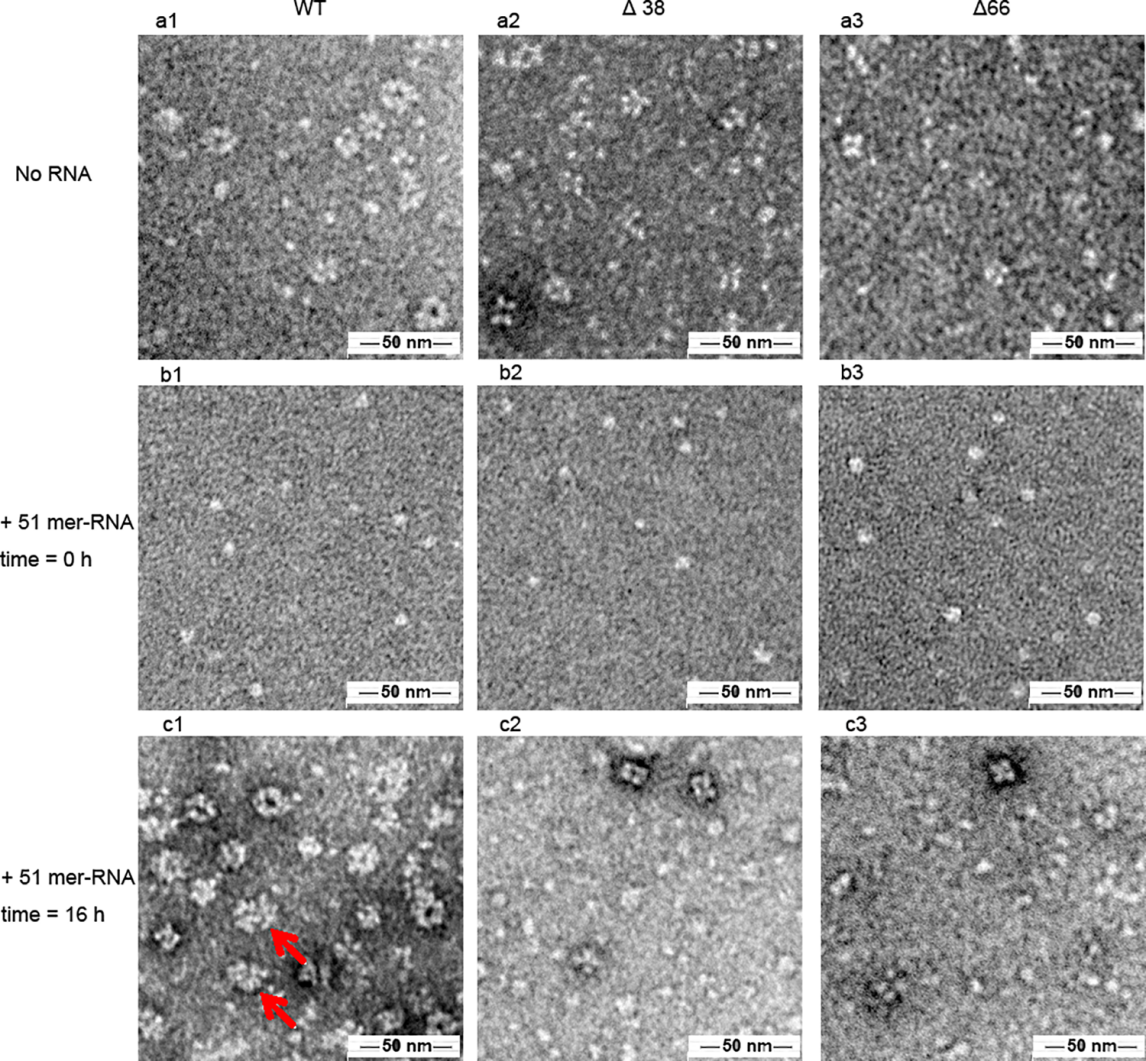
The oligomeric states of wild-type BNP, BNP-Δ38 and BNP-Δ66 examined by election microscopy. Electron microscopy pictures of NP and its variants. *a)* wild-type BNP (*a1*), BNP-Δ38 (*a2)* and BNP-Δ66 (*a3*) in 100 mM sodium phosphate, 100 mM NaCl, pH6.8. b) Wild-type BNP (*b1*), BNP-Δ38 (*b2)* and BNP-Δ66 (*b3*) in PBS when RNA was added at 0h. c) Wild-type BNP (*c1*), BNP-Δ38 (*c2)* and BNP-Δ66 (*c3*) in PBS 16hr after RNA was added. The red arrow in the Fig 3B-c1 indicates the RNA-BNP complexes that displays a double layered structure.

To investigate if the N-terminal region of BNP takes part in oligomerization, BNP wild-type and variants were purified and the oligomeric states of BNP were visualized by electron microscopy. As shown by Fig 4a1, 4a2 and 4a3, both wild-type BNP and BNP mutants were in their oligomeric states. However the truncated protein was shown to maintain a lower oligomeric state than that of wild-type BNP. Wild-type BNP formed mainly hexamers and higher oligomers (Fig 4a1), but its N-terminal truncation variants formed lower level oligomers, mainly tetramers and pentamers (Fig 4a2 and 4a3).

**Fig 5:**
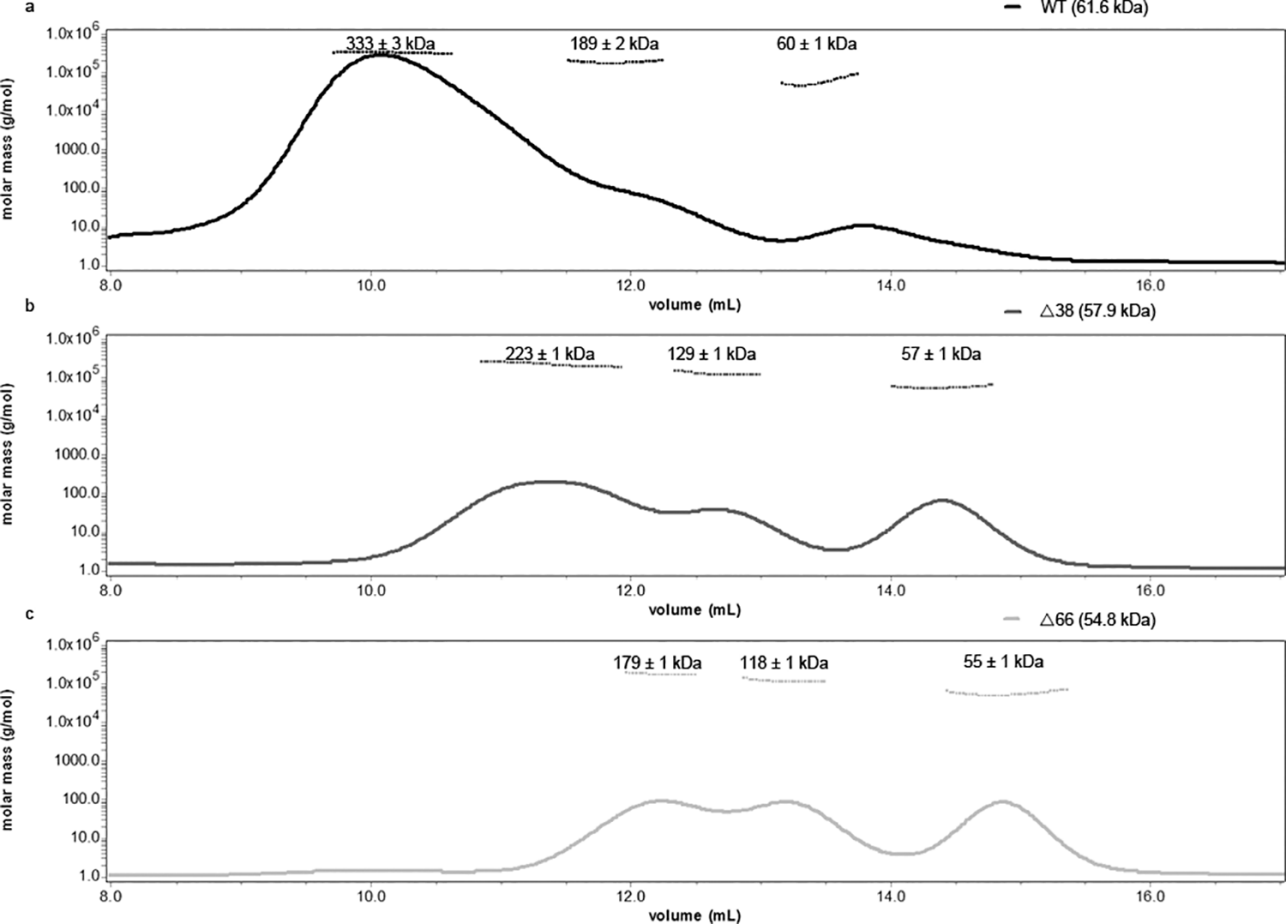
Static light scattering analysis of purified wild-type NP and variants. Horizontal lines on top of the curves illustrate the calculated molecular weights.

Supporting this observation, the light scattering experiment demonstrated that the wild-type BNP formed more oligomers, producing a peak with an average molecular weight of 333 kDa (Fig 5A). In contrast, Δ38 and Δ66 formed more monomers, and the oligomers had molecular weights of 223 and 179 kDa respectively (Fig 5B and 5C). These are approximately 3–4 times the size of a single molecule.

### The N-terminal region of BNP plays a role in the formation of BNP-RNA complex

When incubated with PBS buffer, all BNP constructs were in monomeric states. Monomeric BNP constructs were incubated with 51mer RNA. Upon the addition of RNA, wild-type BNP was induced to form higher oligomers (hexamers and above) after 16 hours (Fig 4c1). Some of the complexes displayed a double layered shape that resembles a part of double helix vRNP structure, as indicated by the red arrows. However the truncated mutants mainly present as scattered monomers. In addition, the RNA-NP oligomers had less NP as compared to the wild-type BNP (Fig 4c2 and 4c3).

**Fig 6:**
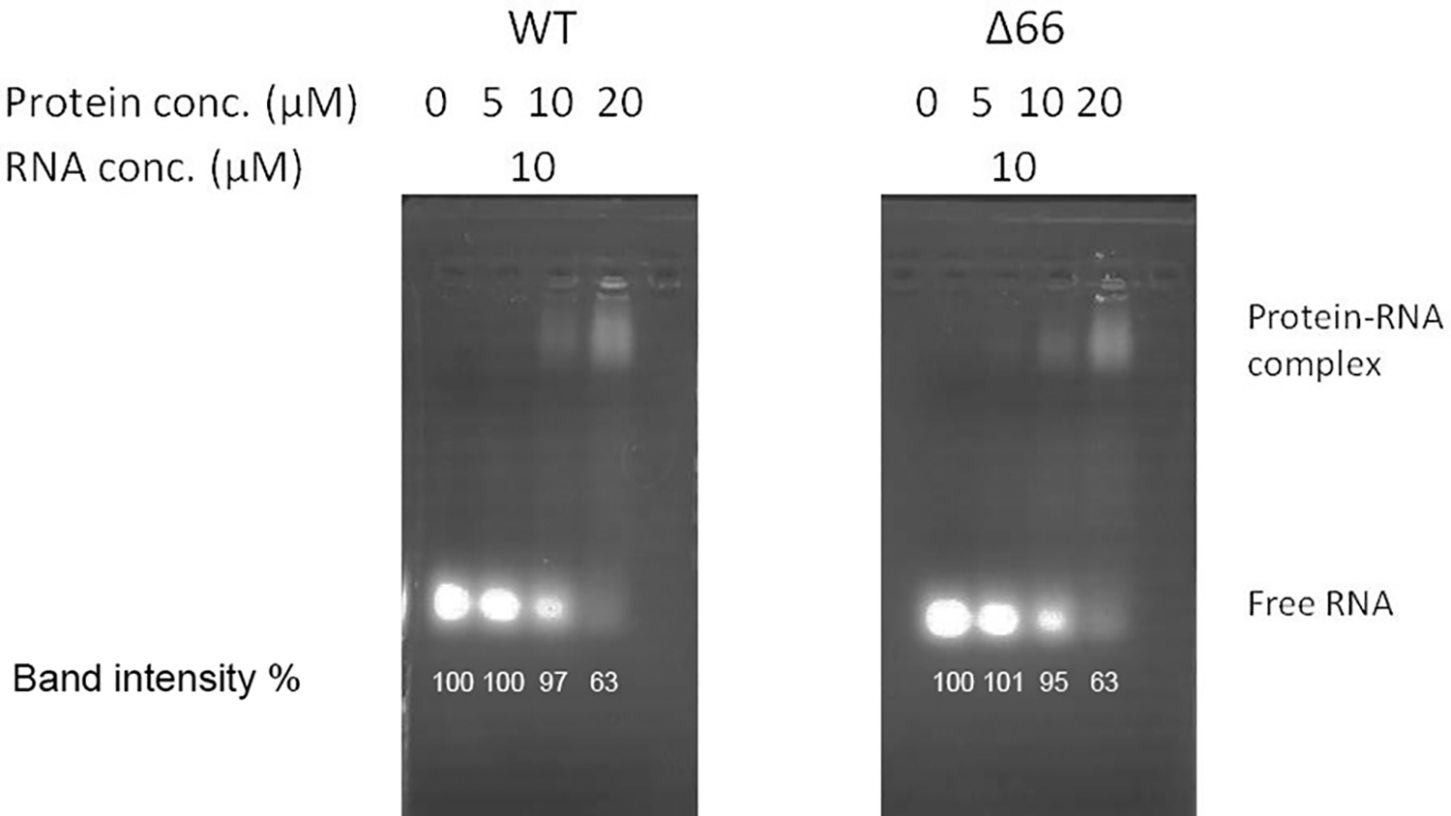
Wild-type BNP and BNP-Δ66 have comparable RNA binding activities. Indicated amount of purified BNP and BNP-Δ66 were incubated with 10 μM of 24 nt 2-O-methylated RNA and analyzed by agarose gel electrophoresis. Band intensities were calculated by ImageJ (National Institutes of Health)[19], using the RNA without the protein as a control (100%).

Gel shift assay was carried out to test the affinity of BNP and its mutants with a 24 mer RNA. It is well known that one ANP binds to about 24 nucleotides of RNA [4]. Based on the structural similarity of BNP and ANP, 24 mer RNA was used to ensure a roughly 1:1 stoichiometry ratio. As shown in Fig 6, wild-type BNP had caused the shift of the RNA band in the concentration of 10 μM. BNP-Δ66 caused a similar effect as wild-type BNP. It is likely that deletion of the N-terminal region did not perturb the RNA binding pocket, which is at aa 125–146 and aa 211–236 and opposite to the N-terminal region[11] and hence the affinity to RNA was not affected.

## Discussion

Nucleoprotein (NP) is one of the major components of the vRNP. It oligomerizes and packs the RNA and functions for transcription and replication of the viral genome [9]. As mentioned before, the nucleoprotein of influenza A and B (ANP and BNP) share many similar characteristics in their structures and functions but differs in the N-terminal region.

We revealed the importance of the N-terminal by virus reconstitution and mini-genome reconstitution. In a well correlated manner, truncations in the N-terminal lead to decrease in vRNP activities and retardation of viral growth. Both recombinant virus and reconstituted vRNP with BNP-Δ38 and BNP-Δ66 are not functional, suggesting an indispensible role of the N-terminus.

We next proceeded to clarify the function of the N-terminal region in detail. The NP variants with NLS at aa 44–47 mutated or deleted were unable to enter the nucleus (Fig 3). The N-terminal region of BNP also facilitates the formation of higher homo-oligomers and RNA-BNP complex, as illustrated by our EM and light scattering assays (Figs 4 and 5). On the other hand, the N-terminal region did not enhance the affinity of BNP to a 24nt RNA.

Previous studies on the N-terminal of BNP have led to different conclusions. This region was first shown to be not required for nuclear import, RNA transcription and replication [20]. However, another group has recently changed K_44_RTR_47_ to A_44_ATR_47_ and showed the NP variant was exclusively cytoplasmic, indicating that this region functions as the NLS of BNP [13]. The N-terminal region of BNP has also been investigated by Sherry and colleagues [14]. In their work, truncation of 11 to 51aa decreased replication in a mini-replicon assay by 30–40% and truncation of more than three amino acids prevented the recovery of virus. They also showed the presence of an NLS K_44_RTR_47_ in the N-terminal region. This sequence is for nuclear localization of BNP but does not take part in RNA transcription [14]. Despite of differences in the fine details, probably due to the use of different flu B strains and assays, our data support the importance of the N-terminal region of BNP in NP transport, RNP activity and viral growth.

Our work also showed that the N-terminal of BNP contributes to the maintenance of higher oligomeric structure and higher level RNA-BNP complexes. On the other hand, truncation of the N-terminal did not fully deprive the ability of BNP in forming oligomers. Lower oligomers can still be formed after deleting this region, whereas truncating the tail loop of ANP and BNP resulted in monomeric NP [10,11]. This suggests that the N-terminal region supports the formation of complexes by stabilizing the formation of high level oligomers. Similar to cases of other negative-sense RNA viruses (e.g. Rabies virus NP[21], vesicular stomatitis virus[22,23], Leanyer virus[24] and Bunyamwera virus[25]), the N-terminal of NP probably binds to neighboring BNP molecules and stabilizes the complex. The lack of this region will hinder the functioning of viral RNP and viral viability, resulting in the inactive of viral RNP and the inability to reconstitute virus with NP deleted 38 and 66aa.

Generally in this study we have highlighted the importance of the N-terminal of BNP for virus viability by participating in the vRNP transcription and replication. This sequence is required to sustain vRNP activity and viral viablity. We have further elucidated that the N-terminal upholds vRNP activity not only by regulating the nuclear entry of BNP. It also has a major contribution on the formation of vRNP by retaining of higher oligomers and forming BNP-RNA complexes. Losing part of these functions may hinder viral growth, resulting in the reduction or failure of virus reconstitution. The study illustrates that the N terminus of BNP is essential for viral viability and this region thus provides with us a novel target for antiviral design.

## Supporting Information

S1 Fig The expression level of pcDNA-BNP variants in 293T cells. The amount of BNP mutants were individually adjusted to have a similar expression level to the wild-type.
(TIF)

## References

[1] Paul Glezen W, Schmier JK, Kuehn CM, Ryan KJ, Oxford J. The burden of influenza B: a structured literature review. Am J Public Health. 2013; 103: e43–51. 10.2105/AJPH.2012.301137 PMC3673513 23327249

[2] Zhao B, Qin S, Teng Z, Chen J, Yu X, Gao Y, et al Epidemiological study of influenza B in Shanghai during the 2009–2014 seasons: implications for influenza vaccination strategy. Clin Microbiol Infect. 2015 Apr 13. pii: S1198-743X(15)00373-0. 10.1016/j.cmi.2015.03.009 25882368

[3] Kawai N, Ikematsu H, Iwaki N, Maeda T, Satoh I, Hirotsu N, et al A comparison of the effectiveness of oseltamivir for the treatment of influenza A and influenza B: a Japanese multicenter study of the 2003–2004 and 2004–2005 influenza seasons. Clin Infect Dis. 2006; 43: 439–444. 16838232 10.1086/505868

[4] Hatakeyama S, Sugaya N, Ito M, Yamazaki M, Ichikawa M, Kimura K, et al Emergence of influenza B viruses with reduced sensitivity to neuraminidase inhibitors. JAMA. 2007; 297:1435–1442. 17405969 10.1001/jama.297.13.1435

[5] Escuret V, Frobert E, Bouscambert-Duchamp M, Sabatier M, Grog I, Valette M, et al Detection of human influenza A (H1N1) and B strains with reduced sensitivity to neuraminidase inhibitors. J Clin Virol. 2008; 41:25–28. 18055253 10.1016/j.jcv.2007.10.019

[6] Hatta M, Kawaoka Y. The NB protein of influenza B virus is not necessary for virus replication in vitro. J Virol. 2003; 77: 6050–6054. 12719596 10.1128/JVI.77.10.6050-6054.2003 PMC154028

[7] Mowshowitz SL, Deval J. Influenza B virus: alpha-amanitin sensitivity of replication and primer-dependence of in vitro transcription. Arch Virol. 1980; 63: 159–163. 7356393 10.1007/BF01320774

[8] Jambrina E, Barcena J, Uez O, Portela A. The three subunits of the polymerase and the nucleoprotein of influenza B virus are the minimum set of viral proteins required for expression of a model RNA template. Virology. 1997; 235: 209–217. 9281500 10.1006/viro.1997.8682

[9] Portela A, Digard P. The influenza virus nucleoprotein: a multifunctional RNA-binding protein pivotal to virus replication. J Gen Virol. 2002; 83: 723–734. 11907320 10.1099/0022-1317-83-4-723

[10] Ng AK, Zhang H, Tan K, Li Z, Liu JH, Chan PK, et al Structure of the influenza virus A H5N1 nucleoprotein: implications for RNA binding, oligomerization, and vaccine design. FASEB J. 2008; 22: 3638–3647. 10.1096/fj.08-112110 18614582 PMC2537428

[11] Ng AK, Lam MK, Zhang H, Liu J, Au SW, Chan PK, et al Structural basis for RNA binding and homo-oligomer formation by influenza B virus nucleoprotein. J Virol. 2012; 86: 6758–6767. 10.1128/JVI.00073-12 22496219 PMC3393550

[12] Yang YH, YST YS, Shaw PC. Structure and function of nucleoproteins from orthomyxoviruses. Biodesign. 2014; 2: 91–99.

[13] Wanitchang A, Narkpuk J, Jongkaewwattana A. Nuclear import of influenza B virus nucleoprotein: involvement of an N-terminal nuclear localization signal and a cleavage-protection motif. Virology. 2013; 443: 59–68. 10.1016/j.virol.2013.04.025 23689061

[14] Sherry L, Smith M, Davidson S, Jackson D. The N terminus of the influenza B virus nucleoprotein is essential for virus viability, nuclear localization, and optimal transcription and replication of the viral genome. J Virol. 2014; 88: 12326–12338. 10.1128/JVI.01542-14 25122787 PMC4248939

[15] Macville M, Schröck E, Padilla-Nash H, Keck C, Ghadimi BM, Zimonjic D. Comprehensive and definitive molecular cytogenetic characterization of HeLa cells by spectral karyotyping. Cancer Res. 1999:59:141–150. 9892199

[16] Jackson D, Cadman A, Zurcher T, Barclay WS. A reverse genetics approach for recovery of recombinant influenza B viruses entirely from cDNA. J Virol. 2002; 76: 11744–11747. 12388735 10.1128/JVI.76.22.11744-11747.2002 PMC136801

[17] Jackson D, Elderfield RA, Barclay WS. Molecular studies of influenza B virus in the reverse genetics era. J Gen Virol. 2011; 92: 1–17. 10.1099/vir.0.026187-0 20926635

[18] Ozawa M, Fujii K, Muramoto Y, Yamada S, Yamayoshi S, Takada A, et al Contributions of two nuclear localization signals of influenza A virus nucleoprotein to viral replication. J Virol. 2007; 81: 30–41. 17050598 10.1128/JVI.01434-06 PMC1797272

[19] Schneider CA, Rasband WS, Eliceiri KW. NIH Image to ImageJ: 25 years of image analysis. Nature Methods. 2012; 9: 671–675. 22930834 10.1038/nmeth.2089 PMC5554542

[20] Stevens MP, Barclay WS. The N-terminal extension of the influenza B virus nucleoprotein is not required for nuclear accumulation or the expression and replication of a model RNA. J Virol. 1998; 72: 5307–5312. 9573310 10.1128/jvi.72.6.5307-5312.1998 PMC116436

[21] Albertini AA, Wernimont AK, Muziol T, Ravelli RB, Clapier CR, Schoehn G, et al Crystal structure of the rabies virus nucleoprotein-RNA complex. Science 2006; 313: 360–363. 16778023 10.1126/science.1125280

[22] Green TJ, Zhang X, Wertz GW, Luo M. Structure of the vesicular stomatitis virus nucleoprotein-RNA complex. Science. 2006; 313: 357–360. 16778022 10.1126/science.1126953

[23] Rainsford EW, Harouaka D, Wertz GW. Importance of Hydrogen Bond Contacts between the N Protein and RNA Genome of Vesicular Stomatitis Virus in Encapsidation and RNA Synthesis. Journal of Virology. 2010; 84: 1741–1751. 10.1128/JVI.01803-09 20007268 PMC2812390

[24] Niu F, Shaw N, Wang YE, Jiao L, Ding W, Li X, et al Structure of the Leanyer orthobunyavirus nucleoprotein-RNA complex reveals unique architecture for RNA encapsidation. Proc Natl Acad Sci U S A. 2013; 110: 9054–9059. 10.1073/pnas.1300035110 23569220 PMC3670306

[25] Ariza A, Tanner SJ, Walter CT, Dent KC, Shepherd DA, Wu WN, et al Nucleocapsid protein structures from orthobunyaviruses reveal insight into ribonucleoprotein architecture and RNA polymerization. Nucleic Acids Research. 2013; 41: 5912–5926. 10.1093/nar/gkt268 23595147 PMC3675483

