# Supplementary figures and images for: The Functional Study of the N-Terminal Region of Influenza B Virus Nucleoprotein
Source: PLoS One. 2015 Sep 14;10(9):e0137802. doi: 10.1371/journal.pone.0137802 (PMC4569402; doi:10.1371/journal.pone.0137802)

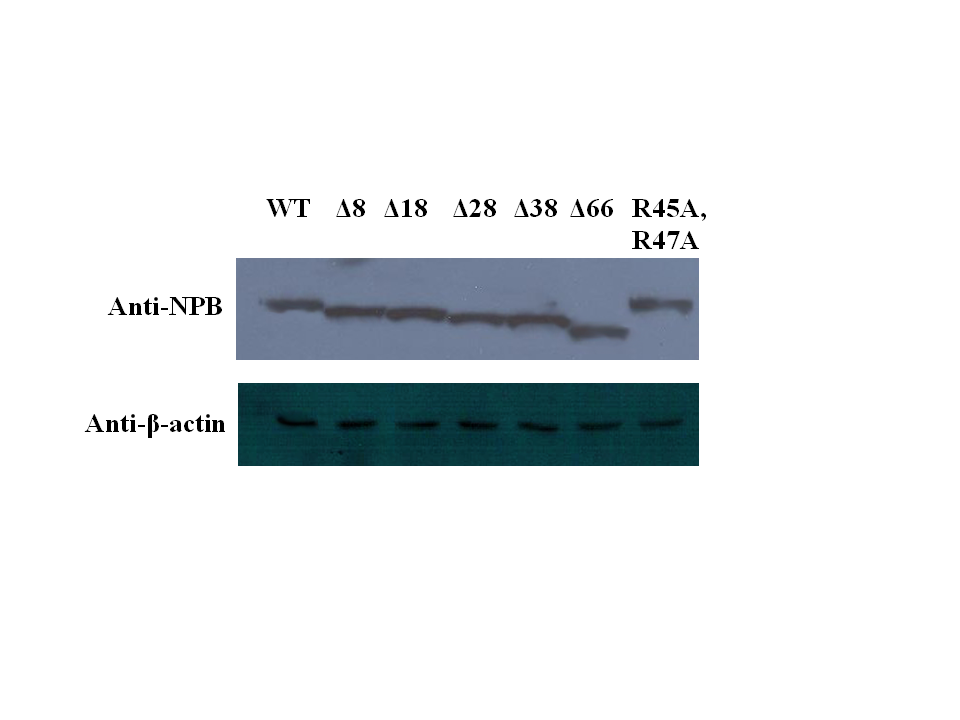

Supplement: S1 Fig — The amount of BNP mutants were individually adjusted to have a similar expression level to the wild-type. (TIF) [file pone.0137802.s001.tif]
